# Quantification and health risk assessment of heavy metals in milled maize and millet in the Tolon District, Northern Ghana

**DOI:** 10.1002/fsn3.1714

**Published:** 2020-06-14

**Authors:** Kate‐Vera Larsen, Samuel J. Cobbina, Samuel A. Ofori, Divine Addo

**Affiliations:** ^1^ Department of Ecotourism and Environmental Management Faculty of Natural Resource and Environment University for Development Studies Tamale Ghana; ^2^ Department of Biology of Organisms Faculty of Science Université Libre de Bruxelles Brussels Belgium; ^3^ Department of Environmental Science College of Science Kwame Nkrumah University of Science and Technology Kumasi Ghana

**Keywords:** disk attrition mill, heavy metals, metal leach, mill plates, milled millet and maize

## Abstract

This study was conducted to quantify the levels and measure the health risk of selected heavy metals (Fe, Zn, Pb, Mn, Cr, Ni) in milled millet and maize from 10 communities in the Tolon District, northern region of Ghana. Control samples of maize and millet were also prepared in order to identify whether the sources of the heavy metals were limited to the milling process or otherwise. The heavy metals present in the collected milled samples were quantified using an AAS Varian Spectra 220 FS. The average concentrations of the selected heavy metals in the milled maize samples were the following: Fe = 1.3392 ± 0.4341 mg/kg, Ni = 0.9502 ± 0.2696 mg/kg, Pb = 2.2177 ± 0.0534 mg/kg, Cr = 0.4359 ± 0.3574 mg/kg, Zn = 0.6809 ± 0.0534 mg/kg, and Mn = 0.3550 ± 0.1042 mg/kg. Milled millet samples recorded mean concentration of the heavy metals as Fe = 1.9467 ± 1.0597 mg/kg, Ni = 0.9520 ± 0.1218 mg/kg, Pb = 2.2780 ± 0.0221 mg/kg, Cr = 0.3421 ± 0.1498 mg/kg, Zn = 0.8540 ± 0.1139 mg/kg, and Mn = 0.4241 ± 0.0859 mg/kg. All selected heavy metals concentrations were below standards in food except Pb. Locally made mill plates were found to leach heavy metals in the milled flours due to the direct contact of the grains with the mill plates. However, the comparison of milled to pounded flour (control samples) indicated other potential sources of heavy metals aside from the disk attrition mill. The health risk assessment revealed no potential hazards nor cancer risk in both children and adults.

## Graphical abstract

This research investigated the levels of some heavy metals in milled grains (maize and millet). Among the heavy metals selected, Pb recorded a concentration higher the permissible limit. Health risk assessment did not indicate risk of cancer for consumers.

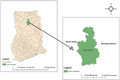

## 1 INTRODUCTION

Cereals are staple foods in most populations of the world due to the relative cheap methods of production and storage. Maize for instance is cultivated over a range of agro‐climatic zones, and its productivity in varying environments makes it second to none among cereals produced in Ghana. Maize consumption rate in Ghana has increased steadily over the years and stands at a rate of about 2,480,000 MT (MOFA, 2016). Millet production in Ghana is able to reliably thrive on marginal lands with low rainfall conditions, making this crop native of the northern regions of Ghana (Kanton et al., 2015). The four types of millets that are cultivated on a large scale are pearl, foxtail, proso, and finger millet; however, pearl and finger millet are typical of Ghana. Several delicacies can be prepared from maize and millet such as porridge, kenkey, “tuo‐zaafi,” desserts (grilled maize), and “tom brown” and drinks such “fula,” “zom koom,” and “zirkom” (Ghana Tourism Authority, 2016).

Food processing methods which include the grinding of coarse food items into smaller sizes have over centuries past been evolving depending on the increase in demand for processed food items. Today, milling is the predominant method of achieving a more digestible form of maize and millet grains in the rural areas (Oniya et al., 2018). The disk attrition mill or corn mill, locally called “Nika Nika,” in Ghana is a typical product made by small‐scale foundries in Ghana by a method known as sand casting. The foundry industry in the country is characterized by workers with little or no knowledge of the chemistry of the metal constituents of their products (Andrews & Gikunoo, 2011). Therefore, their products such as the mill plates in milling machines are noted to be of poor quality and wear off faster, yet many mill operators prefer the locally manufactured disk plates because it is relatively cheap and readily available (Kwofie, Andrews, & Mensah, 2011).

The corn mill uses metallic disks with uniformly rough surface made of ridges, contained in a circular chamber. There are two metallic disks in every corn mill: one is stationery and the other rotating. Friction force where compression and shear forces are applied enables grinding of the grains. The metallic disks of the attrition mill manufactured by the foundry industry in Ghana are usually nonalloyed, made from gray cast iron (Kwofie et al., 2011; Oniya et al., 2018). Studies conducted on heavy metal contamination in milled grains have related the levels of these chemicals to the milling process, that is, leaching of heavy metals like iron (Fe), cadmium (Cd), copper (Cu), lead (Pb), and zinc (Zn) from the metallic disks into the flour produced (Adeti, 2015). Other studies also make conclusions that the farming methods of these cereals have a level of contribution to the increased concentrations of these heavy metals in the grains (Abebe & Chandravanshi, 2017).

The extent of health effects of heavy metal exposure depends on the type and form of the element, route of exposure (ingestion, dermal contact, and inhalation), duration of exposure (acute or chronic), and individuals’ susceptibility (Vardhan, Kumar, & Panda, 2019). Health‐related issues with heavy metal exposure include damage or malfunction of the central nervous activities, lungs, liver, kidneys, circulatory system, larynx, prostate glands, and other fundamental organs (Mahurpawar, 2015; Vardhan et al., 2019).

In the Tolon district, the most preferred form of milling among the indigenes is dry milling and majority of the meals consumed in this area are products of milled maize and millet. This research therefore sought to unravel the levels of Fe, Mn, Ni, Pb, Cr, and Zn present in milled grains of maize and millet and further assess the associated potential health risks posed to their consumers.

## 2 MATERIALS AND METHODS

### 2.1 Study area

**FIGURE 1:**
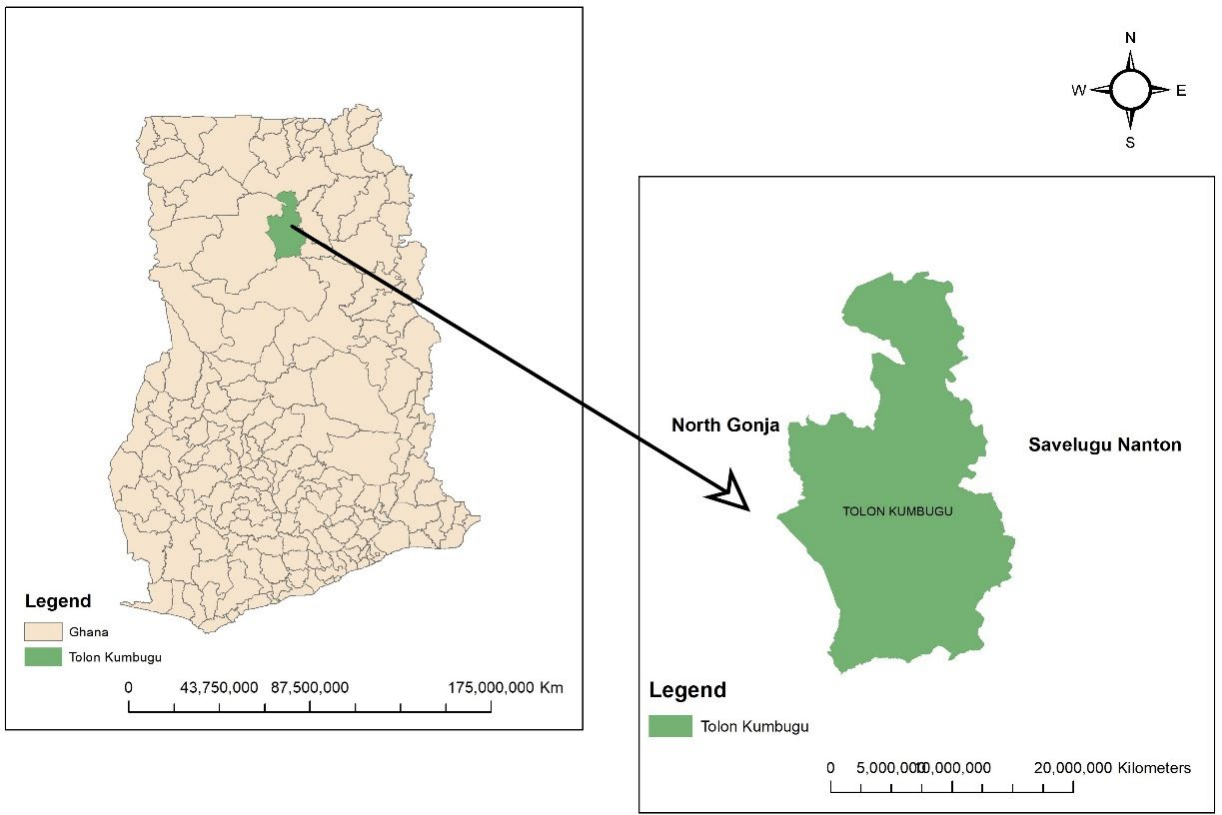
Study area

The research was conducted in the Tolon district, which has a geographical location of 9.6772°N, 1.0203°W. The area records a single rainfall season in each year, contributing to its grassland vegetation interspersed with guinea savannah woodland vegetation. The soil type in the area is usually sandy loam except in the lowlands where alluvial deposits exist. The indigenes of this district are mostly crop farmers at the sustenance level. The Tolon district according to the 2010 Populations and Housing Census consists of twenty large communities, 10 out of twenty communities were randomly selected for this research; they include Chirifoyili, Gbulahgu, Kpendua, Tali, Tingoli, Tolon, Nyankpala, Yipelgu, Wantugu, and Yoggu. (GSS, 2014). The communities were labeled A‐J, respectively (Figure 1).

### 2.2 Sample collection and preparation

From each of the ten selected communities, two (one raw and one milled) samples each of corn and millet were respectively collected before and after milling from the women at the popular corn milling stations during the first and second of weeks in March 2019. The cereals were milled repeatedly to obtain very fine flour at the commercial milling sites. A clean plastic ladle was used in fetching raw and milled samples into low‐density polyethene ziplock bags at sampling point. The raw samples were pounded using clean wooden mortar and pestle and sieved in a 2‐mm mesh in the laboratory in order to obtain a very fine particle size of samples to serve as control. All samples were labelled accordingly and stored in a freezer for chemical analysis.

### 2.3 Sample digestion and analysis

The samples were dried in an oven for 30 min. One gram of each sample was weighed into a filter paper, which was transferred into test tubes. Two tablets of catalyst and 15 ml of H_2_SO_4_ were added to the test tube containing the samples; this was allowed to sit in the digester for 6 hr. The samples were then filtered into plastic wash bottles. An atomic absorption spectrophotometer, Varian Spectra 220 FS, was used in determining the levels of Fe, Mn, Ni, Pb, Cr, and Zn in the digested samples.

### 2.4 Health risk assessment

To determine the potential effects of the heavy metal contamination in samples to the health of both adults and children, the following health risk indicators were calculated for the following:

(1) $$\text{ADD}=\frac{\text{Cm}\times \text{IR}\times \text{EF}\times \text{ED}}{\text{BW}\times \text{AT}}\times 10^{-6}$$

(2) $$\text{HQ}=\frac{\text{ADD}}{\text{RfD}}$$

(3) HI=∑HQ

(4) CancerRisk=SF×ADD

where ADD is average daily dose, Cm is concentration of metal, IR is ingestion rate, EF is exposure frequency, ED is exposure duration, BW is body weight, AT is average time, HQ is hazard quotient, RfD is reference dose, HI is hazard index, and SF is slope factor.

The ADD for both children and adult population via ingestion was evaluated using Equation 1. The cancer and noncancer risks assessment was done using the HQ, HI, and cancer risk approaches as indicated in Equations 2,3 and 4. The HQ refers to the mean daily dose of metal with reference to its reference dose. According to USEPA (2011), HQ and HI values >1 indicate a potential adverse cancer effect, while when <1 denotes a nonadverse cancer effect (Table 1).

**TABLE 1:** Standard values in health risk assessment

Heavy Metals	RfD	SF	Factor	UNIT	Value	
					Children	Adults
Fe	7.0 × 10^–1^		BW	Kg	15	70
Pb	3.5 × 10^–3^	8.5 × 10^–3^	ED	Years	6	63
Ni	2.0 × 10^–2^		EF	days/year	365	365
Cr	3.0 × 10^–3^		AT	Days	ED × 365	ED × 365
Mn	1.4 × 10^–1^		IR	mg/kg	200	100
Zn	3.0 × 10^–1^					

Note

(USEPA, 1989, 2011; WHO, 2018).

## 3 RESULTS AND DISCUSSIONS

The effect of grinding mill on the concentrations of some selected heavy metals in milled maize and millet samples was studied. Chemical analysis of milled samples presented levels of selected heavy metals ranging from 0.3550 ± 0.1042 mg/kg to 2.2177 ± 0.0534 mg/kg for maize and 0.3421 ± 0.1498 mg/kg to 2.2780 ± 0.0221 mg/kg for millet. Among the six selected heavy metals, Pb recorded the highest mean concentration in both maize (2.2177 ± 0.0534 mg/kg) and millet (2.2780 ± 0.0221 mg/kg) samples. Levels of Pb were significantly higher in milled millet than in milled maize (Table 2). The recorded levels of Pb in milled samples were eleven times higher when compared with the permissible limit set by the Codex Alimentarius Commission (CAC) in 2011 (Table 3). When compared to a study done in Iraq on the levels of Pb in milled wheat, the concentration of Pb recorded was four times lower than recorded in this study (Jawad and Allafaji, 2012). Another study conducted on the level of Pb in milled maize recorded lower concentrations of Pb in samples (0.210 ± 0.15 mg/kg) when compared to the levels recorded in this study (Adeti, 2015). This result can be attributed to the metal leaching incident during the milling process when the two metallic disks in the corn mill come into contact. The high levels of Pb recorded in both milled maize and millet samples bring to light an environmental issue of concern as Pb is reported as a persistent, bio‐accumulative, and toxic (PBT) chemical enlisted in the USEPA (United States Environmental Protection Agency) priority list of chemicals (Check & Marteel‐Parrish, 2013). It is therefore essential to note that a continual consumption of milled maize and millet products can lead to an increased exposure to Pb. When Pb enters the cells of a living organism, it can bioaccumulate and thus causes toxic effects. Studies conducted on some animals and humans revealed that continual exposure to Pb caused cancer of the bladder, stomach and lungs, reproductive defects, increased brain and heart problems, anemia, and other health issues (Rehman, Fatima, Waheed, & Akash, 2017).

**TABLE 2:** Statistical test of selected heavy metals in milled maize and millet

Heavy metal	Maize	Millet	*p* < .05
	Mean	Mean	
Ni	0.9502 ± 0.2696	0.9520 ± 0.1218	.9849
Pb	2.2177 ± 0.0534	2.2780 ± 0.0221	.004
Fe	1.3392 ± 0.4341	1.9467 ± 1.0597	.1107
Cr	0.4359 ± 0.3574	0.3421 ± 0.1498	.4539
Zn	0.6809 ± 0.0534	0.8540 ± 0.1139	.0004
Mn	0.3550 ± 0.1042	0.4241 ± 0.0859	.1231

**TABLE 3:** Comparison of selected heavy metals levels in samples with their regulatory standards in food

Heavy metal	Maize (mg/kg)	Millet (mg/kg)	Standard	Regulating bodies
Ni	0.9502	0.9520	10	WHO/FAO, 2001
Pb	2.2177	2.2780	0.2	CAC, 2011
Fe	1.3392	1.9467	15	CAC, 1985
Cr	0.4359	0.3421	1.3	WHO/FAO, 2001
Zn	0.6809	0.8540	30	CAC, 1985
Mn	0.3550	0.4241	2.3	WHO/FAO, 2001

Considering manganese (Mn), relatively lower levels were recorded in milled maize and millet with no significant difference. The levels of Mn recorded in milled maize and millet were 5 and 6 times lower, respectively, when compared with the permissible limits stated by the WHO/FAO (World Health Organization/Food and Agriculture Organization) in 2001 (Table 3). Mn its natural state occurs with other chemicals such as sulfur, oxygen, and chlorine in order to be water soluble. It is enlisted as the 12th most abundant element in the earth crust, and the 5th most abundant metal. Even though this heavy metal is ubiquitous in the environment, it is recorded in low levels in environmental samples; food, air, water, and soil (Rōllin & Nogueira, 2011). This affirms the low levels of Mn recorded in the milled maize and millet samples. Similar studies conducted by Abrefah, Mensimah, Sogbadji, and Opata (2011) in Accra metropolis, Ghana, reported records of Mn concentration in milled maize being lower than the recommended levels in food. Studies have indicated that Mn intake at low levels can serve as essential nutrients necessary for healthy cartilage and bone formation, mitochondria protection, and glucose production in the human body (Agency for Toxic Substances & Disease Registry, 2012). Even though Mn levels in the environment can be low and essential for catalytic and regulatory functions in the human body, it is revealed that a continual increased exposure to this chemical can cause central nervous system disorders (Rōllin & Nogueira, 2011).

Nickel (Ni) levels recorded in milled samples were 10 times lower when compared with the permissible limits indicated by the WHO/FAO in, 2001. The relatively low levels of Ni recorded in milled samples can be associated with its low levels in the environment. Nickel is considered as the 24th most abundant element in the earth crust (Rehman et al., 2017). Like Mn, low concentrations of Ni are needed in the human body for vital functioning of certain organs, and it is however unsafe when taken into the body at high and increased levels. Ni contamination has been reported to majorly occur in food products as it is used together with its alloys in manufacturing food processing machines (Sharma, 2013). A study conducted by Abrefah et al. (2011) on milled maize samples collected from milling joints in Accra Metropolis, Ghana, recorded Ni concentrations ranging from 26.18 ± 3.23 mg/kg to 46.42 ± 2.53 mg/kg. The high levels recorded in this study were attributed to the duration of milling process, as a longer period of milling increases the friction occurring between the two plates of the corn mill machine. Low levels of Ni and Mn recorded in milled samples of the current study make it safe for the consumption of milled maize and millet, indicating lower chances of the development of health defects such as lung, kidney, and oral cancer in consumers.

The levels of iron (Fe), zinc (Zn) and chromium (Cr) recorded in the milled samples were all lower than their respective permissible limits defined by the Codex Alimentarius Commission (CAC) (1985) and WHO/FAO (2001). In their low levels of intake into the human body, Fe, Zn, and Cr are considered as essential for the proper functioning of organs and systems. Fe is considered as the second most abundant metal and as such an essential element necessary for the synthesis of blood pigments and other essential cell processes (Reilly, 2002). Zn is also identified as a useful element that is relevant for regulation of gene expression, cell growth, and differentiation in living organisms like humans. Although Cr is a widely occurring element on earth, it is reported to be at relatively low levels. Since the 1950s, Cr has been identified as an essential element for humans due to its important role in promoting the metabolism of carbohydrates and lipids in the body, thereby maintaining blood glucose levels, especially in diabetic patients (Onakpa, Anoka, & Ogbureke, 2018; Reilly, 2002). Like other essential metals, an intake of higher and increased concentrations of Fe, Zn, and Cr via ingestion of contaminated food will lead to the development of toxic effects such as haemochromatosis and clastogenesis (chromosome breakdown), respectively (Reilly, 2002). The relatively low levels of these metals recorded in the milled samples certifies that it is safe for one to consume milled products of maize and millet since the milling process does not contribute much to the levels of these metals in samples.

**FIGURE 2:**
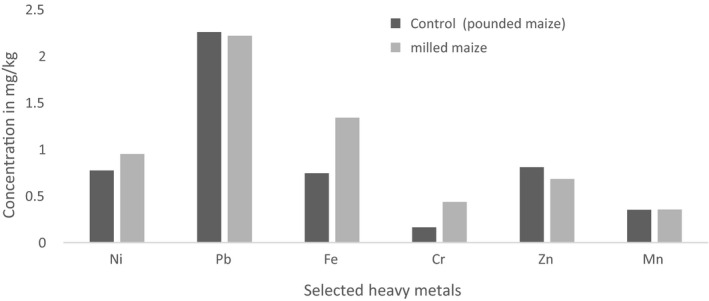
Mean concentrations of heavy metals in milled maize and control maize

In order to identify the actual or net contribution of milling process on the levels of heavy metals recorded in the milled samples, a control group of samples were developed by pounding raw maize and millet grains separately in a wooden mortar and pestle. Considering the milled maize samples, the mean concentrations of Fe (1.34 mg/kg), Cr (0.44 mg/kg), Ni (0.95 mg/kg), and Mn (0.36 mg/kg) recorded were higher as compared to their corresponding concentrations in the control samples. Only Zn (0.81 mg/kg) and Pb (2.26 mg/kg) recorded a lower mean concentration in milled maize samples than in their corresponding concentrations in the control samples (Figure 2).

**FIGURE 3:**
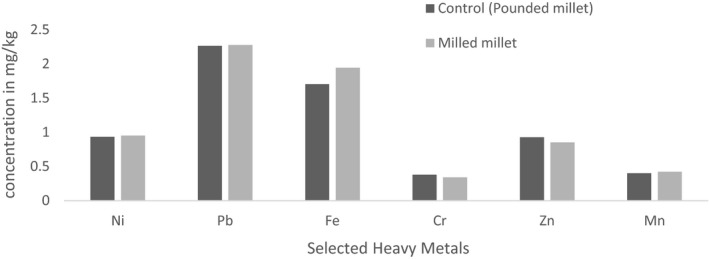
Mean concentrations of heavy metals in milled millet and control millet

For milled millet samples, the mean concentrations of Fe (1.95 mg/kg), Mn (0.42 mg/kg), Ni (0.95 mg/kg), and Pb (2.29 mg/kg) were found to be higher than their corresponding recorded levels in the control samples. For Cr and Zn, higher mean concentrations were recorded in control samples (0.382 mg/kg and 0.928 mg/kg, respectively) than in the milled millet samples (0.342 mg/kg and 0.854 mg/kg, respectively) (Figure 3).

The increased concentrations of Fe, Cr, Ni, and Mn in milled maize samples and Fe, Mn, Ni, and Pb in milled millet samples in comparison with their corresponding levels in control samples could be attributed to the leaching of metals arising from the friction of the milling plates. This is affirmed by a study conducted by Kalagbor, Fyneface, Korfii, Ogaji, and Kpoonanyie (2017) who also reported the levels of Fe in milled maize to be higher than in control samples. Also, a study conducted by Israila and Halima (2016) in Nigeria reported higher levels of Fe and Mn in milled samples of maize than in control samples.

For the metals Pb and Zn, they were found to be of higher levels in the control samples than in the milled samples. Similar to this result were the higher levels of Zn recorded in control samples which were reported by Abrefah Mensimah Sogbadji and Opata (2011) in their study on the levels of some selected heavy metals in milled and pounded maize samples in Accra, Ghana. According to Chilian, Bancuta, Bancuta, and Setnescu (2015), maize and millet plants are tolerant to Zn which helps to promote their growth and yield. This has a close relation to the Zn levels in the soil that is readily taken up by plants. It is also noteworthy that Zn levels in millet are naturally high; however, their level can be further increased by manure application in pasture lands especially as concluded by Wang, Wang, Kou, Ma, and Zhao (2016). A reduced concentration of Zn in both milled samples could therefore be attributed to its interaction with other metals such as Cu which has the ability to oxidize elemental Zn to Zinc oxide (ZnO) in the presence of water (VMZINC, n.d). This describes the antagonistic effect of Cu on Zn and thus confirms why milled maize samples recorded lower levels of Zn.

The higher levels of Pb recorded in the control samples of maize could be ascribed to the situation of soil contamination due to continuous tillage which is typical of savannah soils (Agbenin, 2002) and other possible sources of heavy metal pollution such as effluents from industrial activities (Malomo, Olufade, Adekoyeni, & Jimoh, 2013). In contrast, Israila and Halima (2016) in Nigeria recorded higher levels of Pb in milled samples of maize than in control samples. It is however unclear what may have caused the reduced Pb levels in both milled maize and millet samples except interactive processes with other metals during the milling process leading to the formation of new chemicals which were not analyzed in this study.

Millet control samples recorded a higher concentration of Cr and Zn than the corresponding milled samples. An increase in Cr and Zn concentrations present in the millet control samples could be attributed to farming or other soil contamination activities such as pesticide and inorganic fertilizer application. A study by Srinivasarao (2014) revealed that the phosphate‐based fertilizers were produced from phosphate rocks containing heavy metals such as Cr, Pb, Ni, and Zn. A reduced level of Cr in the milled millet samples could be determined by possible chemical reaction with Fe during the milling process, thereby causing the formation of stainless steel (Jonsson et al., 2016) (Table 4).

**TABLE 4:** Average daily dose (mg/kg) of heavy metals in milled maize for adults and children

Community	Nickel		Lead		Iron		Chromium		Zinc		Manganese	
	Adults	Children	Adults	Children	Adults	Children	Adults	Children	Adults	Children	Adults	Children
A	1.157 × 10^–6^	1.080 × 10^–5^	3.126 × 10^–6^	2.917 × 10^–5^	1.571 × 10^–6^	1.467 × 10^–5^	2.643 × 10^–7^	2.467 × 10^–6^	9.714 × 10^–7^	9.067 × 10^–6^	4.129 × 10^–7^	3.853 × 10^–6^
B	1.043 × 10^–6^	9.733 × 10^–6^	3.253 × 10^–6^	3.036 × 10^–5^	1.719 × 10^–6^	1.604 × 10^–5^	1.034 × 10^–6^	9.653 × 10^–6^	9.514 × 10^–7^	8.880 × 10^–6^	4.457 × 10^–7^	4.160 × 10^–6^
C	1.231 × 10^–6^	1.149 × 10^–5^	3.173 × 10^–6^	2.961 × 10^–5^	1.593 × 10^–6^	1.487 × 10^–5^	3.329 × 10^–7^	3.107 × 10^–6^	9.686 × 10^–7^	9.040 × 10^–6^	4.286 × 10^–7^	4.0000 × 10^–6^
D	1.156 × 10^–6^	1.079 × 10^–5^	2.989 × 10^–6^	2.789 × 10^–5^	1.783 × 10^–6^	1.664 × 10^–5^	2.886 × 10^–7^	2.693 × 10^–6^	1.146 × 10^–6^	1.069 × 10^–5^	8.757 × 10^–7^	8.173 × 10^–6^
E	1.277 × 10^–6^	1.192 × 10^–5^	3.160 × 10^–6^	2.957 × 10^–5^	1.643 × 10^–6^	1.533 × 10^–5^	3.757 × 10^–7^	3.507 × 10^–6^	9.714 × 10^–7^	9.067 × 10^–6^	4.329 × 10^–7^	4.040 × 10^–6^
F	2.310 × 10^–6^	2.156 × 10^–5^	3.213 × 10^–6^	2.999 × 10^–5^	2.309 × 10^–6^	2.155 × 10^–5^	1.520 × 10^–6^	1.419 × 10^–5^	9.557 × 10^–7^	8.920 × 10^–6^	6.614 × 10^–7^	6.173 × 10^–6^
G	1.209 × 10^–6^	1.128 × 10^–5^	3.214 × 10^–6^	3.000 × 10^–5^	1.471 × 10^–6^	1.373 × 10^–5^	2.443 × 10^–7^	2.280 × 10^–6^	9.557 × 10^–7^	8.920 × 10^–6^	4.314 × 10^–7^	4.027 × 10^–6^
H	1.751 × 10^–6^	1.635 × 10^–5^	3.247 × 10^–6^	3.031 × 10^–5^	2.073 × 10^–6^	1.935 × 10^–5^	1.444 × 10^–6^	1.348 × 10^–5^	8.286 × 10^–7^	7.733 × 10^–6^	4.814 × 10^–7^	4.493 × 10^–6^
I	1.149 × 10^–6^	1.072 × 10^–5^	3.157 × 10^–6^	2.947 × 10^–5^	1.464 × 10^–6^	1.367 × 10^–5^	2.357 × 10^–7^	2.200 × 10^–6^	9.914 × 10^–7^	9.253 × 10^–6^	4.143 × 10^–7^	3.867 × 10^–6^
J	1.291 × 10^–6^	1.205 × 10^–5^	3.141 × 10^–6^	2.932 × 10^–5^	3.506 × 10^–6^	3.272 × 10^–5^	4.871 × 10^–7^	4.547 × 10^–6^	9.871 × 10^–7^	9.213 × 10^–6^	4.871 × 10^–7^	4.547 × 10^–6^

The average daily dose (ADD) of selected heavy metals for adults and children who consume the milled maize in the 10 selected communities in the Tolon district was determined and represented. Fe recorded the highest ADD (3.506 × 10^–6^ and 3.272 × 10^–5^ mg/kg) for adults and children, respectively, in community J, while the least ADD for adults was recorded by Cr in community G (2.443 × 10^–7^ mg/kg) and that of the children was recorded in community I with an ADD value of 2.200 × 10^–6^ mg/kg (Tables 5 and 6).

**TABLE 5:** Average daily dose (mg/kg) of heavy metals in milled millet for adults and children

Community	Nickel		Lead		Iron		Chromium		Zinc		Manganese	
	Adults	Children	Adults	Children	Adults	Children	Adults	Children	Adults	Children	Adults	Children
A	1.737 × 10^–6^	1.621 × 10^–5^	3.244 × 10^–6^	3.028 × 10^–5^	2.047 × 10^–6^	1.911 × 10^–5^	9.814 × 10^–7^	9.160 × 10^–6^	1.289 × 10^–7^	1.203 × 10^–5^	6.343 × 10^–7^	5.920 × 10^–6^
B	1.487 × 10^–6^	1.388 × 10^–5^	3.303 × 10^–6^	3.083 × 10^–5^	1.680 × 10^–6^	1.568 × 10^–5^	7.429 × 10^–7^	6.933 × 10^–6^	9.771 × 10^–7^	9.120 × 10^–6^	5.200 × 10^–7^	4.853 × 10^–6^
C	1.199 × 10^–6^	1.119 × 10^–5^	3.206 × 10^–6^	2.992 × 10^–5^	1.536 × 10^–6^	1.433 × 10^–5^	2.771 × 10^–7^	2.587 × 10^–6^	1.287 × 10^–7^	1.201 × 10^–5^	4.900 × 10^–7^	4.573 × 10^–6^
D	1.414 × 10^–6^	1.320 × 10^–5^	3.231 × 10^–6^	3.016 × 10^–5^	3.620 × 10^–6^	3.379 × 10^–5^	4.643 × 10^–7^	4.333 × 10^–6^	1.440 × 10^–7^	1.344 × 10^–5^	7.271 × 10^–7^	6.787 × 10^–6^
E	1.427 × 10^–6^	1.332 × 10^–5^	3.281 × 10^–6^	3.063 × 10^–5^	1.246 × 10^–6^	1.163 × 10^–5^	4.300 × 10^–7^	4.013 × 10^–6^	1.303 × 10^–7^	1.216 × 10^–5^	5.014 × 10^–7^	4.680 × 10^–6^
F	1.291 × 10^–6^	1.205 × 10^–5^	3.303 × 10^–6^	3.083 × 10^–5^	2.973 × 10^–6^	2.775 × 10^–5^	3.986 × 10^–7^	3.720 × 10^–6^	9.686 × 10^–7^	9.040 × 10^–5^	6.971 × 10^–7^	6.507 × 10^–6^
G	1.159 × 10^–6^	1.081 × 10^–5^	3.249 × 10^–6^	3.032 × 10^–5^	3.859 × 10^–6^	3.601 × 10^–5^	4.014 × 10^–7^	3.747 × 10^–6^	1.214 × 10^–7^	1.133 × 10^–5^	5.471 × 10^–7^	5.107 × 10^–6^
H	1.314 × 10^–6^	1.227 × 10^–5^	3.247 × 10^–6^	3.031 × 10^–5^	1.323 × 10^–6^	1.235 × 10^–5^	4.471 × 10^–7^	4.173 × 10^–6^	1.120 × 10^–6^	1.045 × 10^–5^	4.757 × 10^–7^	4.440 × 10^–6^
I	1.391 × 10^–6^	1.299 × 10^–5^	3.237 × 10^–6^	3.021 × 10^–5^	3.523 × 10^–6^	3.288 × 10^–5^	4.471 × 10^–7^	4.173 × 10^–6^	1.181 × 10^–7^	1.103 × 10^–5^	6.157 × 10^–7^	5.747 × 10^–6^
J	1.180 × 10^–6^	1.101 × 10^–5^	3.241 × 10^–6^	3.025 × 10^–5^	6.004 × 10^–6^	5.604 × 10^–5^	2.971 × 10^–7^	2.773 × 10^–6^	1.420 × 10^–6^	1.325 × 10^–5^	8.500 × 10^–7^	7.933 × 10^–6^

**TABLE 6:** Hazard index for children and adults

Heavy metal	Children	Adults
Ni	8.500 × 10^–2^	5.212 × 10^–3^
Pb	1.998 × 10^–3^	9.160 × 10^–3^
Fe	3.129 × 10^–3^	2.260 × 10^–3^
Cr	5.187 × 10^–3^	2.320 × 10^–3^
Zn	5.847 × 10^–2^	3.518 × 10^–3^
Mn	1.484 × 10^–4^	4.488 × 10^–5^

Considering milled millet samples, Fe again recorded the highest ADD (3.859 × 10^–6^ and 3.601 × 10^–5^ mg/kg) in community G for adults and children respectively, while the least ADD was recorded by Zn in community H for adults (1.120 × 10^–6^ and mg/kg) and that of children was recorded by Cr in community J (2.773 × 10^–6^ mg/kg).

From the estimated ADD of selected heavy metals in the various communities, it can therefore be deduced that both adults and children were exposed more to Fe than the other heavy metals present in the milled maize and millet which they averagely consumed daily. Cr and Zn were recorded as the heavy metals with the least ADD among the communities in the consumption of milled maize and millet. It could also be deduced that consumers of the milled maize and millet in the communities would face the issue of deficiency in Cr and Zn which is at some level is considered essential for their health.

The hazard quotient values obtained in this study were all <1. Also, the hazard index (HI) values of the selected heavy metals for both children and adults were less than 1 for each heavy metal present in the milled maize and millet. According to USEPA (2011), values >1 indicate potential adverse effect, and values <1 would denote a nonadverse effect to the health of the consumers. All the HQ and HI values recorded in this study were all below one; therefore, the results in this study reveal there are no adverse effect upon consumption of milled maize and millet. This is in contrast with the research conducted in Kumasi, Ghana by Adeti (2015), which recorded the HI values of Cr (1.438) and Ni (1.65) to be >1 in the first milled maize samples using locally made plates.

## 4 CONCLUSION

The levels of all the selected heavy metals were below WHO/FAO standards except Pb, which was above the permissible limit of 0.2 mg/kg. This indicated a health concern regarding the consumption of both raw and milled samples of maize and millet in the region. However, low levels of the other heavy metals recorded provided a degree of safety in the consumption of maize and millet. The potential sources of the heavy metal contaminants in this study range from operational activities of milling machine (sharpening, welding, alignment of mill plates, and painting), soil contamination, and application of phosphate‐based fertilizer and pesticides. The health risk assessment shows there is no potential health risk for both adults and children who consume milled maize and millet. Nevertheless, the health risk of consumption of milled maize and millet in this region cannot be over emphasized since there exists poor milling operational activities and sustenance farming is prevalent, indicating contained soil contamination, which may lead to an increased contamination over a long period.

## 5 ETHICAL REVIEW

This study does not involve any human or animal testing.

## CONFLICT OF INTEREST

The authors declare that they do not have any conflict of interest.

## INFORMED CONSENT

Written informed consent was obtained from all study participants.
